# Distribution des dermatoses rencontrées chez les enfants vus en consultation dermatologique à Lomé (Togo)

**DOI:** 10.48327/mtsi.v2i2.2022.243

**Published:** 2022-06-10

**Authors:** Julienne Noude TÉCLESSOU, Kokoè Afiwa DOVI-TEVI, Koussake KOMBATÉ, Bayaki SAKA, Abla Séfako AKAKPO, Palokinam PITCHÉ

**Affiliations:** 1 Service de dermatologie, Centre hospitalier universitaire de Lomé, Faculté des sciences de la santé, Université de Lomé, Togo; 2 Service de dermatologie, Centre hospitalier universitaire Sylvanus Olympio, Faculté des sciences de la santé, Université de Lomé, Togo

**Keywords:** Distribution, Dermatoses immunoallergiques, Dermatoses infectieuses, Enfants, Hôpital, Lomé, Togo, Afrique subsaharienne, Distribution, Immunoallergic dermatoses, Infectious dermatoses, Children, Hospital, Lomé, Togo, Sub-Saharan Africa

## Abstract

**Objectif:**

Le but de cette étude était d’établir le panorama des dermatoses chez les enfants vus en consultation dermatologique à Lomé et de décrire les tendances de ces dermatoses entre 1992 et 2020.

**Méthode:**

Il s'agit d'une étude rétrospective descriptive portant sur les dossiers des patients de 0 à 15 ans vus en consultation dans les services publics de dermatologie de Lomé du 1er janvier 2010 au 31 décembre 2019. Une comparaison des différentes dermatoses a été faite avec celles recensées lors d'une précédente étude similaire en 1992.

**Résultats:**

Au cours de la période d’étude, 3 767 enfants ont consulté pour une affection dermatologique (14,2 % de consultations pédiatriques parmi les consultations dermatologiques). L’âge moyen des patients était de 7,4 ans et le sexe ratio M/F était de 0,7. Les mois de juillet, août et septembre étaient ceux qui enregistraient le plus de consultations. Les trois premiers motifs de consultation étaient l'eczéma (26,6%), le prurigo strophulus (15,3%) et la dermatite atopique (4,9%). Aussi, 51,3 % des pathologies cutanées chez les enfants étaient représentées par les dermatoses immunoallergiques, suivies des dermatoses infectieuses (23,6%). Les dermatoses infectieuses prédominantes étaient mycosiques (34,4%) et bactériennes (30,3%). On note entre 1992 et 2019 une augmentation de la prévalence des dermatoses immunoallergiques et une réduction des dermatoses infectieuses.

**Conclusion:**

Les résultats de cette étude montrent que les dermatoses immunoallergiques sont prédominantes et en nette augmentation chez les enfants vus en consultation dermatologique à Lomé. Nous l'attribuons à l'urbanisation et aux changements climatiques.

## Abstract (en)

**Objective:**

The aim of this study was to establish the overview of current dermatosis in children in dermatological consultation in Lomé and to deduce the evolutionary trend of these dermatoses between 1992 and 2020.

**Method:**

This was a retrospective descriptive study concerning patients aged 0 to 15 years seen in consultation in the public dermatological services of Lomé from January 1, 2010 to December 31, 2019. The reasons for consultation were reviewed and classified into groups of dermatosis (immunoallergic, infectious, inflammatory, tumoral …). The results of this series were compared with those of a similar study carried out in 1992.

**Results:**

During the study period, 3 767 children attended showing dermatological condition (14.2% of the dermatological consultations were pediatric). The average age of the patients was 7.4 years and the sex ratio M/F was 0.7. July, August and September were the months with the highest number of consultations. The top three reasons for consultation were eczema (26.6%), prurigo strophulus (15.3%) and atopic dermatitis (4.9%). 51.3% of skin diseases in children were represented by immunoallergic dermatoses, followed by infectious dermatoses 23.6%. The predominant infectious dermatoses were fungal (34.4%) and bacterial (30.3%). Between 1992 and 2019, there was an increase in the prevalence of immunoallergic dermatoses and a reduction in infectious dermatoses.

**Conclusion:**

Immunoallergic dermatoses are predominant and clearly increasing among children seen in dermatological consultations in Lomé.

## Introduction

La pathologie cutanée de par sa morbi-mortalité est considérée comme un problème de santé publique aussi bien dans les pays en voie de développement que dans les pays développés [7, 11]. Elle semble être sous-estimée car elle ne motive une consultation que si elle occasionne une gêne fonctionnelle ou un préjudice esthétique important [9]. Plusieurs études ont porté sur les motifs de consultation en dermatologie tant chez les adultes que chez les enfants en Afrique subsaharienne [3, 5, 9]. Les dermatoses chez les enfants représentent 20 % à 31,5 % des motifs de consultations selon différentes études en Afrique [1,2,4,6]. Les dermatoses couramment rapportées chez les enfants en Afrique subsaharienne sont d'origine infectieuse (29,2 % à 55,1%), qu'il s'agisse de consultations dermatologiques ou d’études réalisées dans la population générale infantile [2, 4, 6, 12]. En 1992 au Togo [12], une étude portant sur les motifs de consultation chez les enfants avait noté respectivement 36,4 % et 34,11 % de dermatoses allergiques et infectieuses chez les enfants de 0 à 5 ans. Compte tenu de l'urbanisation et du développement des villes, une variation du profil des dermatoses chez l'enfant pouvait être suspectée.

Le but de cette étude est d’établir le panorama des dermatoses chez les enfants en consultation dermatologique à Lomé et d'estimer la tendance de ces dermatoses entre 1992 et 2020.

## Patients et Méthode

Il s'est agi d'une étude rétrospective descriptive sur une période de 10 ans (1^er^ janvier 2010 au 31 décembre 2019) dans les services de dermatologie des centres hospitaliers universitaires Sylvanus Olympio, Campus et au centre de dermatologie de Gbossimé. Les enfants vus en consultation dans l'un des trois services de dermatologie pendant la période d’étude ont été inclus. Nous avons considéré les enfants de 0 à 15 ans. Les dossiers médicaux des patients ont été revus. Les variables étudiées étaient sociodémographiques (âge, sexe) et clinique (diagnostic retenu).

Les motifs de consultation sont exprimés en groupe : dermatoses immunoallergiques (eczéma, dermatite atopique, prurigo strophulus, urticaires, lupus …), dermatoses infectieuses (bactériennes, virales, mycosiques, parasitaires), dermatoses inflammatoires (acné, pityriasis rosé de Gibert.).

Les différentes dermatoses ont été comparées avec celles recensées lors de la précédente étude.

## Résultats

Sur les 26 554 patients vus en consultation dermatologique pendant la période d’étude, 3 805 (14,32%) étaient des enfants de 0 à 15 ans. Parmi ces derniers, 48 dossiers étaient inexploitables. Nous avons donc retenu 3 767 (14,2%) enfants présentant une dermatose pour notre étude. Les enfants consultaient pour une ou plusieurs dermatoses. En effet, 2 à 3 dermatoses étaient diagnostiquées chez 161 (4,3%) des 3767 enfants.

Pendant les 10 années de consultation, les mois d'août sont ceux où l'on observe la fréquence la plus élevée des consultations (591 soit 15,7%), suivis respectivement des mois de juillet (510 soit 13,53%) et de septembre (446 soit 11,8%).

Les cinq premiers motifs de consultation étaient : l'eczéma de contact (1 002 cas soit 26,6%), le prurigo strophulus (615 soit 16,3%), la dermatite atopique (186 soit 4,9%), les folliculites et furoncles (172 soit 4,5%), et la scabiose (155 soit 4,1%). L’âge moyen des patients était de 7,4 ans +/- 5,5 (extrêmes : 1 jour et 15 ans). L’âge médian était de 7 ans et le sexe ratio (M/F) de 0,7.

Plus de la moitié (1 933 soit 51,3%) des pathologies cutanées ayant fait l'objet d'une consultation par les enfants de 0-15 ans était représentée par les dermatoses immunoallergiques, suivies des dermatoses infectieuses 890 (23,6%). Le taux des dermatoses immunoallergiques était quasi constant de 2010 à 2019, allant de 994 (51,4%) à 945 (48,9%). Les dermatoses infectieuses varient de 41 (22,7%) en 2010 à 157 (27,5%) en 2019 (Tableau I). En fonction des mois, les dermatoses immunoallergiques connaissent des pics pendant les mois juillet, août et septembre allant de 221 à 309 cas (Tableau I). Il en est de même pour les dermatoses infectieuses avec des pics de 115 à 122 pendant les mois de juillet, août et septembre.

**Tableau I:** Variation des différents groupes de dermatoses selon les mois et les années (Lomé, Togo, 2010-2019) Variation of the different groups of dermatosis according by month and year (Lomé, Togo, 2010-2019)

	DIA *N(%)	Dermatoses Infectieuses N(%)	Dermatoses Inflammatoires N(%)	IST** N(%)	Dermatoses Tumorale N(%)	Autres dermatoses N(%)	Total N(%)
**Mois**							
Janvier	108 (2,9)	51 (1,4)	18 (0,5)	0 (0,0)	4 (0,1)	30 (0,7)	211 (5,6)
Février	99 (2,6)	33 (0,8)	16 (0,4)	0 (0,0)	4 (0,1)	29 (0,7)	181 (4,8)
Mars	121 (3,2)	45 (1,2)	10(0,2)	0 (0,0)	3 (0,07)	27 (0,5)	206 (5,5)
Avril	106 (2,8)	51 (1,3)	20 (0,5)	0 (0,0)	6 (0,2)	32 (0,8)	215 (5,7)
Mai	157 (4,1)	74 (1,9)	27 (0,7)	4 (0,1)	14 (0,3)	40 (1,0)	316 (8,4)
Juin	159 (4,2)	58 (1,5)	27 (0,5)	2 (0,05)	5 (0,1)	34 (0,9)	285 (7,6)
Juillet	253 (6,7)	122 (3,2)	50 (1,3)	0 (0,0)	10 (0,2)	75 (2,0)	510 (13,5)
Aout	309 (8,2)	117 (3,1)	66 (1,7)	1 (0,02)	15 (0,4)	83 (2,2)	591 (15,7)
Septembre	221 (5,8)	115 (3,0)	37 (1,0)	2 (0,05)	9 (0,2)	62 (1,6)	446 (11,8)
Octobre	148 (3,9)	71 (1,8)	21 (0,5)	1 (0,02)	6 (0,2)	35 (0,9)	282 (7,5)
Novembre	133 (3,5)	75 (1,9)	23 (0,6)	1 (0,02)	5 (0,1)	19 (0,5)	256 (6,8)
Décembre	119 (3,1)	78 (2,0)	34 (0,9)	1 (0,02)	6 (0,2)	30 (0,8)	268 (7,1)
**Total**	**1933 (51,3)**	**890 (23,6)**	**349 (9,3)**	**12 (0,3)**	**87 (2,3)**	**496 (13,1)**	**3767 (100)**
**Année**								
2010	93 (51,4)	41 (22,7)	18 (9,9)	0 (0,0)	2 (1,1)	27 (14,9)	181 (4,8)
2011	172 (51,8)	65 (19,5)	36 (10,8)	2 (0,6)	4 (1,2)	53 (16,0)	332 (8,8)
2012	127 (55,2)	48 (20,9)	25 (10,9)	1 (0,4)	4 (1,7)	25 (10,9)	230 (6,1)
2013	146 (48,2)	68 (22,4)	28 (9,2)	0 (0,0)	3 (1,0)	58 (19,1)	303 (8,0)
2014	69 (53,9)	20 (15,7)	15 (11,7)	0 (0,0)	3 (2,3)	21 (16,4)	128 (3,4)
2015	39 (40,2)	26 (26,9)	11 (11,3)	1 (1,0)	4 (4,1)	16 (16,5)	97 (2,6)
2016	67 (44,7)	36 (24,0)	18 (12,0)	0 (0,0)	4 (2,7)	25 (16,7)	150 (4,0)
2017	690 (54,3)	319 (25,0)	83 (6,5)	3 (0,2)	31 (2,4)	145(11,4)	1271 (33,7)
2018	251 (49,8)	110 (21,8)	55 (10,9)	2 (0,4)	17 (3,4)	69 (13,7)	504 (13,4)
2019	279 (48,9)	157 (27,5)	60 (10,5)	3 (0,5)	15 (2,6)	57 (10,0)	571 (15,1)
**Total**	**1933 (51,3)**	**890 (23,6)**	**349 (9,3)**	**12 (0,3)**	**87 (2,3)**	**496(13,1)**	**3767 (100)**

* Dermatoses immunoallergiques

** Infections sexuellement transmissibles

Les dermatoses immunoallergiques étaient prédominantes chez les patients âgés de 31 mois à 5 ans (330 soit 63,1%) et chez les nourrissons de 1 à 30 mois (546 soit 59,7%) alors que les dermatoses infectieuses étaient prédominantes chez les patients de moins de 1 mois (40%) (Tableau II). Les dermatoses immunoallergiques étaient plus fréquentes chez les patients de sexe féminin (1 125 soit 58,2%).

**Tableau II:** Répartition des groupes de dermatoses infantiles en fonction des tranches d’âge (Lomé, Togo, 2010-2019) Distribution of pediatric dermatosis groups according to age groups (Lomé, Togo, 2010-2019)

Groupes de dermatoses	Tranches d’âge					
	< 1 mois	1-30 mois	31 mois-5 ans	6-10 ans	11-15 ans	Total
	N (%)					
DIA*	3 (20)	546 (59,7)	330(63,1)	401 (47,0)	653 (44,7)	1933(51,3)
**Dermatoses infectieuses**	6 (40)	256 (27,9)	125(24)	212 (24,9)	291(19,9)	890 (23,6)
**Dermatoses inflammatoires**	1 (6,7)	10 (1,1)	11(2,1)	88 (10,3)	239 (16,3)	349 (9,3)
**Dermatoses tumorales**	2 (13,3)	12 (1,3)	11 (2,1)	26 (3,0)	36 (2,5)	87 (2,3)
IST**	0 (0,0)	5 (0,5)	0 (0,0)	1 (0,1)	6(0,4)	12 (0,3)
**Autres dermatoses**	3 (20)	85 (9,3)	46 (8,8)	125 (14,7)	237 (16,2)	496 (13,2)
**Total**	15 (100)	914 (100)	523 (100)	853 (100)	1462 (100)	3767 (100)

* Dermatoses immunoallergiques

** Infections sexuellement transmissibles

Les dermatoses immunoallergiques retrouvées chez les enfants de 0 à 15 ans étaient essentiellement de l'eczéma de contact représentant 1006 soit 52 % de l'ensemble des dermatoses immunoallergiques, suivis du prurigo strophulus (615 soit 31,8%) et de la dermatite atopique (186 soit 9,6%) (Tableau III).

Les dermatoses infectieuses, deuxième groupe de dermatoses fréquemment retrouvées chez 890 enfants (23,6%), étaient essentiellement représentées par les infections mycosiques (307 soit 34,4%) et bactériennes (270 soit 30,3%). Les dermatoses mycosiques étaient plus fréquentes chez les enfants de sexe masculin (179 soit 58,3%). Les teignes représentaient la majorité des infections mycosiques chez l'enfant (106 soit 34,5%), suivies du pityriasis versicolor (92 soit 30%) (Tableau III). La tranche d’âge des 6-10 ans était plus atteinte par les teignes (73 soit 68,4%).

**Tableau III:** Dermatoses observées chez les enfants en consultation dermatologique à Lomé (Togo, 2010-2019) Dermatoses observed in children seen in dermatology in Lomé (Togo, 2010-2019)

Dermatoses	N	%
**Dermatoses immunoallergiques (DIA)**	1 933	51,3
eczéma de contact	1 006	52,0
prurigo strophulus	615	31,8
dermatite atopique	186	9,6
eczématide	39	2,0
urticaire	36	1,9
**Dermatoses infectieuses**	890	23,6
**mycosiques**	307	34,4
teigne	106	34,5
pityriasis versicolor	92	30,0
dermatophytie circinée	82	26,7
**bactériennes**	270	30,3
folliculites / Furoncles	172	63,7
impétigo	81	30,0
érysipèle	6	2,2
ecthyma	5	1,8
**virales**	101	11,5
molluscum contagiosum	37	36,6
verrues	33	32,7
varicelle	18	17,8
zona	9	8,9
**parasitaires**	212	23,8
scabiose	155	73,2
larbish	56	26,4
**Dermatoses inflammatoires**	349	9,3
pityriasis rosé de Gibert	157	45,0
acné	116	33,2
lichen plan / striatus / nitidus	48	13,8
**Dermatoses tumorales**	87	2,3
chéloïdes	34	41,9
harmatome / Naevus	14	17,9
botriomycome	7	8,6
granulome annulaire	7	8,6
**Infections sexuellement transmissibles (IST)**	12	0,3
condylome	8	66,6
herpès génital	2	16,7
**Autres dermatoses**	496	13,2
kératodermie palmoplantaire	79	15,9
névrodermite / Lichénification	72	14,5
prurit isolé	44	8,9
**Total**	**3 767**	**100**

## Discussion

Cette étude nous a permis d’établir le panorama des dermatoses infantiles vues en consultation dermatologique à Lomé au cours des années 2010 à 2019 et d'en dégager les tendances. Les dermatoses immunoallergiques étaient observées chez plus de la moitié des enfants, suivies des dermatoses infectieuses.

La principale limite de cette étude est sa période. En effet l’étude a porté sur 10 années (2010 à 2019) alors que la précédente étude sur les motifs de consultation chez les enfants à Lomé concernait la seule année 1992. Ceci pourrait engendrer un biais dans la comparaison des dermatoses observées dans la précédente étude avec celles observées dans notre étude. Cependant, les dossiers des patients n’étant pas informatisés dans les différents services de dermatologie, la perte de certains dossiers a justifié ce choix des 10 années. Par ailleurs, les étiologies des dermatoses immunoallergiques n'ont pas été étudiées par manque de données dans les dossiers des patients.

Pendant la période d’étude, 3767 (14,2%) enfants de 0 à 15 ans vus en consultation avaient représenté une dermatose. En 1992 à Lomé, 16,8 % des consultations étaient réalisées chez les enfants de 0 à 15 ans [12]. Nos résultats restent cependant faibles par rapport à ceux rapportés dans la sous-région. En effet, les dermatoses chez les enfants représentaient 24,6 % dans l’étude de Téclessou *et al.* au Centre national hospitalier universitaire de Cotonou au Bénin [13]. En outre, la proportion des enfants vus en consultation dermatologique en milieu hospitalier à Bukavu en République démocratique du Congo (RDC) était de 40,3 % [11].

Pendant les mois de juillet, août et septembre nous avons observé une fréquence élevée de consultation chez les enfants (446 cas soit 11,8 % à 591 soit 15,7%). Ceci peut s'expliquer par le fait que ces mois représentent les vacances scolaires au Togo; où les enfants peuvent être conduits facilement dans les structures publiques de soins sans perturber les heures de cours. Les mêmes observations étaient faites par Semikenke *et al.* au Congo [11].

D'une façon générale, cette étude montre une nette prédominance des dermatoses immunoallergiques chez les enfants vus en consultation dermatologique à Lomé (1933 soit 51,3%). Dans une précédente étude en 1992 à Lomé, une prédominance des dermatoses immunoallergiques était déjà notée chez les enfants bien qu'en proportion moindre (36%) [12]. L'augmentation des dermatoses immunoallergiques en consultation dermatologique à Lomé entre 1992 et 2010-2019 pourrait s'expliquer par une urbanisation de plus en plus importante autour de la ville de Lomé qui est la plus grande ville du pays. En effet, cette étude ayant concerné les services de dermatologie de Lomé, capitale du Togo, la plupart des enfants venant en consultation vivent en zone urbaine et périurbaine. Nous pouvons donc évoquer la transition épidémiologique qui s'accompagne de l'exposition des populations des villes à des allergènes et des polluants, et par conséquent une augmentation progressive des maladies immunoallergiques. Dans une étude réalisée en milieu scolaire chez les enfants en 2019 à Ouagadougou au Burkina Faso [6], les dermatoses immunoallergiques et inflammatoires (29%) étaient le deuxième groupe de dermatose. En 2016 à Bamako au Mali, les dermatoses immunoallergiques venaient au deuxième rang des motifs de consultation chez les enfants (32,5%) après les dermatoses infectieuses (51,1%) [4]. Il serait donc important d’étudier les dermatoses chez les enfants dans la population générale à Lomé et dans d'autres villes du Togo afin d'affirmer ou infirmer cet aspect de la transition épidémiologique.

L'eczéma (1 006 soit 51,8%), le prurigo strophulus (615 soit 31,8%) et la dermatite atopique (186 soit 9,6%) étaient les principales dermatoses immunoallergiques. Dans la précédente étude réalisée par Tchangaï-Walla *et al.* en 1992 à Lomé, l'eczéma (57,9%) et le prurigo strophulus (36,8%) étaient déjà les dermatoses immunoallergiques les plus fréquentes chez les enfants [12].

Le prurigo strophulus chez les enfants est décrit comme une réaction allergique aux piqûres d'insectes, survenant surtout en saison pluvieuse en milieu tropical. Les mois de juillet, août et septembre où nous avons noté des pics de consultation correspondent à la saison pluvieuse au Togo avec une prolifération des insectes piqueurs. À cette période, les eaux de pluie stagnent dans les zones périurbaines peu assainies où prolifèrent les insectes piqueurs. D'où la fréquence élevée du prurigo strophulus.

Cependant ces pics de consultation n’étaient pas liés au seul fait du prurigo puisque les autres dermatoses immunoallergiques et les dermatoses infectieuses avaient également une fréquence élevée au cours de ces mois.

Le nombre élevé d'eczéma (1006 soit 52 % des dermatoses immunoallergiques) dans notre étude peut s'expliquer par l'industrialisation avec comme conséquence une augmentation des allergènes et des sensibilisations. En 2016 au Mali, l'eczéma atopique (15,6%) et le prurigo (9,5%) étaient les dermatoses immunoallergiques les plus fréquentes chez les enfants [4].

Notre étude montre qu'entre 1992 et 2019 la fréquence des dermatoses immunoallergiques a augmenté (de 36 % à 51%), pendant que celle des dermatoses infectieuses a baissé progressivement (de 34 % à 24%) au cours de la même période chez les enfants.

## Conclusion

Les motifs de consultation chez les enfants à Lomé au cours des années 2010-2019 sont dominés par les dermatoses immunoallergiques en nette augmentation 20 ans après la précédente étude. D'où la nécessité de développer des unités de dermato-allergologie pour une meilleure prise en charge de ces patients.

## Liens D'intérêts

Les auteurs ne déclarent aucun lien d'intérêt.

## Contribution Des Auteurs

J. N. Téclessou, K. A. Dovi-Tevi, K. Kombaté : conception de l’étude, rédaction et validation du protocole, collecte des données, rédaction et correction du manuscrit.

B. Saka, A. S. Akakpo, P. Pitché : rédaction et correction du manuscrit.

Tous les auteurs ont lu et approuvé la dernière version du manuscrit.

## References

[1] Adégbidi H, Degboé B, Saka B, Elégbédé A, Atadokpèdé F, Koudoukpo C, Yédomon H, do-Ango Padonou F (2014). Profil des dermatoses immunoallergiques chez les enfants dans le service de dermatologie du CNHU-C (Bénin). Med Sante Trop.

[2] Diabaté A, Kourouma S, Kouabenan AA, Gué I, Vagamon B, Aka BR (2018). Profil épidémiologique, clinique et évolutif des infections parasitaires cutanées superficielles en milieu hospitalier en Côte-d'Ivoire. Rev Int Sci Méd (Abidjan).

[3] Doe PT, Asiedu A, Acheampong JW, Rowland Payne CME (2001). Skin diseases in Ghana and the UK. Int J Dermatol.

[4] Fofana Y, Traoré B, Dicko A, Faye O, Berthe S, Cisse L, Keita A, Tall K, Kone MB, Keita S (2016). Profil épidémio-clinique des dermatoses chez les enfants vus en consultation dermatologique dans le service de dermatologie du Centre national d'appui à la lutte contre la maladie à Bamako (Mali) [Epidemio-clinical profile of dermatoses in children receiving dermatological consultation in the Department of Dermatology at the National Center for Disease Control in Bamako (Mali)]. Pan Afr Med J.

[5] Kobangué L, Lénguébanga F, Dibéré Kamba G, Guéréndo P, Togué A, Abeyé J, Yapoumandji B, Namdito P, Grésenguet G (2014). Étude transversale des affections dermatologiques au service de dermatologie et de vénérologie de Bangui. République Centrafricaine. Rev. CAMES SANTE.

[6] Korsaga/Somé N, Zoungrana/Ouédraogo A, Konaté I, Ouédraogo/Ouédraogo M, Tapsoba GP, Sosso-Kargougou N, Koueta F, Coulibaly A, Andonaba J-B, Niamba P, Barro/Traoré F, Traoré A (2019). Dermatoses en milieu préscolaire dans la ville de Ouagadougou (Burkina Faso) : Aspects épidémiologique, clinique et thérapeutique [Skin disorders in preschool environment in the city of Ouagadougou (Burkina-Faso): Epidemiological, clinical and therapeutic aspects]. Our Dermatol Online.

[7] Lamchahab FE, Beqqal K, Guerrouj B, Khoudri I, Senouci K, Hassam B, Ait Ourhroui M (2010). Bilan d'hospitalisation du service de dermatologie-vénérologie du CHU Ibn Sina Rabat Maroc. Pan Afr Med J.

[8] Lukasiewicz E, Martel J, Roujeau JC, Flahault A (2002). La dermatologie libérale en France métropolitaine en 2000 [Dermatology in private practice in France in 2000]. Ann Dermatol Venereol.

[9] Mahé A, Cissé IAh, Faye O, N'Diaye HT, Niamba P (1998). Skin diseases in Bamako (Mali). Int J Dermatol.

[10] Pitché P, Tchangaï-Walla K (2000). La dermatologie en Afrique Noire. Quelle perspective pour le 21e siècle?. Nouv Dermatol.

[11] Semikenke S, Adégbidi H, Minani J, Bisimwa G (2018). Les dermatoses de l'enfant en milieu hospitalier à Bukavu : aspects épidémiologiques et cliniques. Ann Dermatol Venereol.

[12] Tchangaï-Walla K, Pitché P, Agbèrè A, Bakondé B (1995). Les motifs de consultations des enfants en dermatologie à Lomé (Togo). Med Afr Noire.

[13] Téclessou J, Atadokpédé F, Adégbidi H, Koudoukpo C, Eroume T, N'Dah P, Yédomon HG, do Ango-Padonou F (2011). Motifs de consultation en Dermatologie-Vénéréologie en 2009 à Cotonou, Bénin [Reasons for consultation in a dermatology/venereology department in 2009 in Cotonou, Benin]. Med Trop (Mars).

